# A Mysterious Case of Spontaneous Cervical Epidural Hematoma and Bilateral Primary Spontaneous Pneumothorax caused by a Rare Etiology

**DOI:** 10.5005/jp-journals-10071-23151

**Published:** 2019-04

**Authors:** Keta Thakkar, Neeraja Ajayan, Ajay Prasad Hrishi, Unnikrishnan P, Manikandan Sethuraman

**Affiliations:** 1-5 Neuroanesthesia Division, Department of Anesthesiology, Sree hitra Tirunal Institute for Medical Sciences and Technology, Thiruvananthapuram, Kerala, India

**Keywords:** Primary spontaneous pneumothorax, Spontaneous spinal epidural hematoma, Vasculopathy

## Abstract

Spontaneous spinal epidural hematoma (SSEH) is a rare disorder that can present as an acute onset of pain and radicular symptoms that mimic disk herniation. Primary spontaneous pneumothorax (PSP) can be defined as presence of air in the pleural space without apparent underlying lung disease or trauma. We describe a rare case of SSEH in cervical spine (C5–7) presenting with a novel association, a bilateral PSP, which has never been documented before. Both SSEH and spontaneous pneumothorax, though rare, can be associated with vasculopathy and our patient had signs of vasculopathy of intracranial vessels and pulmonary vasculature. Vasculopathy also needs to be considered while evaluating a case of PSP without emphysema-like changes (ELCs) and if diagnosed as the cause, pleurodesis could be considered as a part of the management to avoid the sequel of recurrent pneumothorax and prolonged ICU stay in this subset of patients.

**How to cite this article** Thakkar K, Ajayan N, *et al*. A Mysterious Case of Spontaneous Cervical Epidural Hematoma and Bilateral Primary Spontaneous Pneumothorax caused by a Rare Etiology. Indian J Crit Care Med 2019;23(4):186–187.

## INTRODUCTION

Spontaneous spinal epidural hematoma (SSEH) is a rare disorder that can present as an acute onset of pain and radicular symptoms that mimic disk herniation. Primary spontaneous pneumothorax (PSP) can be defined as presence of air in the pleural space without apparent underlying lung disease or trauma. We describe a rare case of SSEH in cervical spine (C5-7) presenting with a novel association, a bilateral PSP, which has never been documented before.

## CASE HISTORY

**Fig. 1:**
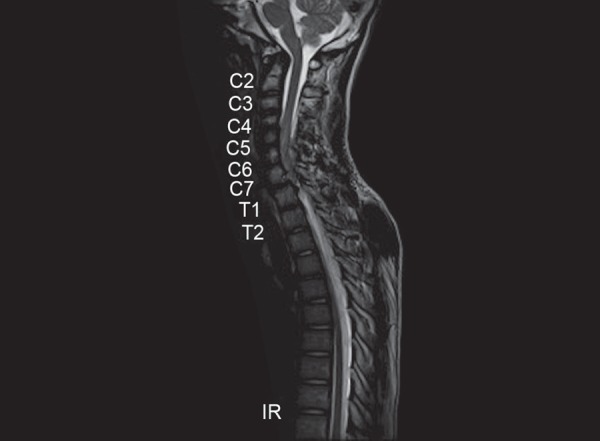
Magnetic resonance imaging (MRI) spine and brain—extradural lesion of size 4.5 × 0.8 cm at C5–7 with cord compression and edema.

An 18-year-old male presented with complaints of an acute onset of severe neck pain and weakness of bilateral lower limbs and upper limbs. The patient was normal until 16 hours prior to admission when he had an acute onset of paresthesia followed by weakness of bilateral lower limbs which rapidly ascended to involve bilateral upper limbs over 6 hours. There was no history of an antecedent trauma, fever or significant family history. On neuromuscular examination, he had grade 0/5 power in bilateral lower limbs, 2/5 in upper limbs with 10% grip and sensory loss below T5 level. Magnetic resonance imaging (MRI) spine and brain revealed an extradural lesion measuring 4.5 × 0.8 cm at C5–7 with cord compression and edema with two intramuscular lesions, measuring 3 × 4 cm and 4 × 3.5 cm in the right paravertebral region (Fig. 1). No abnormalities were detected in brain imaging and autoimmune work up. Patient underwent an emergency cervical (C5–6) laminectomy and hematoma evacuation and postdecompression the cord was found to be pulsatile. Patient was extubated postoperatively and there was gradual improvement in the upper limb power. A postoperative digital subtraction angiography of brain and spine vasculature revealed multifocal areas of luminal irregularity and narrowing, with characteristic beaded appearance suggestive of vasculopathy. MR vessel weighted imaging (MRVWI) of the brain and spine cord revealed minimal enhancement and thickening of mutilple vessels with no ischemic changes or microhemorrhages, suggestive of vasculopathy.

**Fig. 2:**
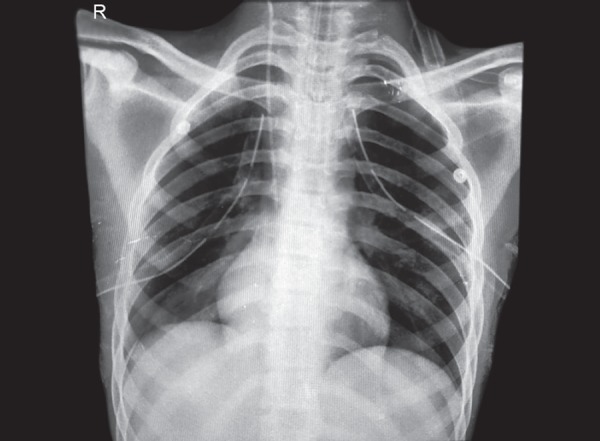
Chest X-ray anteroposterior (AP) view showing bilateral intercostal draining chest tubes.

On postoperative day 15, patient developed a sudden onset of breathlessness with desaturation which warranted emergency intubation. On auscultation, there was absent air entry on left side of the chest and chest X-ray (CXR) revealed a left sided pneumothorax for which left intercostal drainage (ICD) tube was inserted in left hemithorax. Patient improved symptomatically and was extubated two days later with ICD in situ. The extubation trial was unsuccessful as patient again developed respiratory distress and was reintubated and ventilated. A repeat CXR showed a development of right sided pneumothorax which was treated by right sided ICD. High-resolution computed tomography (HRCT) of chest failed to demonstrate any parenchymal or pleural blebs but revealed thickening of the vascular lumen possibly suggestive of pulmonary vasculopathy (Fig. 2). On day two patient was extubated with bilateral ICD in-situ. After 48 hours the ICDs were removed following which patient again developed respiratory distress and was reintubated. Repeat CXR revealed multiple air pockets/pneumothorax of left lung and ICD was reinserted. Serial CXRs revealed similar presentation in the right lung also. In view of the recurrent pneumothorax, successful pleurodesis of both lungs were done and the ICDs were removed.

## DISCUSSION

Spontaneous spinal epidural hematoma is defined as an epidural hematoma occurring without any history of trauma or iatrogenic procedure with an incidence of 0.1 per 100,000 people.^1,2^ SSEHs often present with an abrupt onset of severe neck or back pain that can radiate into the extremities and progresses toward paraparesis or quadriparesis, depending on the level of the lesion. MRI of spine is the imaging modality of choice and once confirmed warrants urgent surgical intervention.^2^ The majority of spinal hematomas (29.7%) have no etiological factor and few cases have been related to anticoagulant therapy and vascular malformations. Our patient had no history of trauma or other precipitating factors and no coagulation abnormality. Additionally, no vascular malformations were observed during routine MRI or CT. DSA and MRVWI demonstrated features suggestive of vasculopathy. Vasculopathy commonly affects the central nervous system (CNS) resulting in CNS pathologies and spinal cord involvement is usually rare.

Pneumothorax, is a clinical emergency that requires immediate intervention. Primary spontaneous pneumothorax is a rare scenario which is defined as the presence of air in the pleural space without apparent underlying lung pathology or trauma.^3–5^ Most clinicians believe that PSP is caused by spontaneous rupture of emphysema-like changes (ELCs), i.e. bullae or subpleural bleb.^5^ Further research into PSP has revealed alternative explanations. It is proposed that there are areas of “altered pleural porosity” which are the site of air leakage. In these areas, there is disruption of the mesothelial cells of the visceral pleura which gets replaced by an elastofibrotic layer caused by inflammatory/ischemic response resulting in increased porosity, allowing air to leak into the pleural space.^3,5^ These areas are predominantly seen in the upper lung zones but not necessarily restricted to apical areas and can often be present bilaterally.^5,6^ Alternative routes of air leakage such as an alveolar rupture into the peribronchovascular interstitium can also result in PSP.^5^ In our patient, the cause of bilateral PSP could be attributed to the vasculopathy of the pulmonary vasculature resulting in zones of altered pleural porosity. Pulmonary vasculopathy, with pulmonary artery medial hypertrophy and intimal fibrosis of pulmonary vessels, has not been reported as a cause of bilateral PSP. Therapeutic intervention by pleurodesis either chemical or mechanical may be sufficient in patients in whom no clearly leaking ELC are visible in CXR, CT or thoracoscopy. Injection of a sclerosing agent via chest tube is an effective therapeutic option in patients without ELC where the etiology could be attributed to connective tissue disorders or vasculopathy.^6–8^

Both SSEH and spontaneous pneumothorax, though rare, can be associated with vasculopathy and our patient had signs of vasculopathy of intracranial vessels and pulmonary vasculature. Vasculopathy also needs to be considered while evaluating a case of PSP without ELCs and if diagnosed as the cause, pleurodesis could be considered as a part of the management to avoid the sequel of recurrent pneumothorax and prolonged ICU stay in this subset of patients.
